# A Scalable and Secure Group Key Management Method for Secure V2V Communication

**DOI:** 10.3390/s20216137

**Published:** 2020-10-28

**Authors:** Hayotjon Aliev, HyungWon Kim, Sunghyun Choi

**Affiliations:** 1 Department of Electronics Engineering, Chungbuk National University, Cheongju 28644, Korea; hayotjon@cbnu.ac.kr; 2 Samsung Research, Samsung Electronics, Seoul 06765, Korea; sunghyunc.research@gmail.com

**Keywords:** scalable group key management, secure group communication, vehicle-to-vehicle communication, matrix-based group key generation, group message encryption algorithm

## Abstract

Safety applications based on vehicle-to-everything (V2X) communications can significantly enhance road safety and reduce traffic fatalities. Ensuring the security and privacy of the vehicular network is essential for the widespread adoption of V2X communications for commercial use. V2X safety and service applications require periodic broadcast communications among all the vehicles. However, compared to unicast communication, it is extremely challenging to provide broadcast communication with network security requirements such as confidentiality, in infotainment contents distribution, sensor data sharing, and security credentials management services. To address the providing confidentiality of vehicle-to-vehicle (V2V) broadcasting, we propose a group key management and message encryption method that is secure, lightweight, and scalable. The proposed group key management method can efficiently handle various scenarios like a node joining or leaving the group, with scalable rekeying algorithms. It employs a distributed and scalable architecture that offers several advantages such as the reduction of the key management overhead and the enhancement of the security level by keeping the key sizes with large networks. In addition, the proposed method employs a lightweight matrix-based encryption algorithm that can be easily applicable with the proposed group key management method. Further, we have implemented the proposed method and evaluated the performance using a V2V network simulator with several networks of highly dynamic group members. The simulation results show that the proposed method can reduce computation time for group key generation and message encryption by more than 80% compared to existing methods.

## 1. Introduction

Group communication is a special type of communication over wired and wireless networks wherein data are exchanged in the form of broadcast packets. During the last decades, many new technologies and concepts, especially based on the theory of group communication, have been implemented. Due to the flexibility and scalability of group communication, increasing attention has been drawn towards new applications such as vehicular communication, social media, digital media, control systems, billing systems, and infotainment in an Internet of Things (IoT) paradigm.

Vehicular communication technologies have been steadily developing and are recently playing an important role in future autonomous cooperative driving. Vehicle-to-everything V2X represents modern vehicular communication technology and intelligent traffic management for autonomous driving, where X represents anything such as infrastructure, vehicles, pedestrians, and roads. The two leading V2X technologies are long-term evolution-vehicle (LTE-V) technology and dedicated short-range communications (DSRC) technology. To exchange credentials and security information among the vehicles within the network, vehicles communicate through vehicle-to-vehicle (V2V) and vehicle-to-infrastructure (V2I) communication [1]. In V2V communication, vehicles communicate with neighboring vehicles mostly via broadcast messages, whereas in V2I communication, vehicles communicate with roadside units (RSUs) or base stations via unicast messages.

The security problem of our concern is the establishment and maintenance of a secure V2X communication via broadcast messages in a dynamic and distributed key management fashion. In this paper, the V2X broadcast communication is referred to as V2X group communication. Recent studies have proposed several approaches for enhancing the V2X group communications security with improved availability, authentication, integrity, and nonrepudiation. However, only few solutions have been reported for confidentiality and privacy problems in V2X group communications, while their importance is rapidly growing. Therefore, ensuring data confidentiality and privacy in V2X group communications is considered a major obstacle for the widespread deployment of V2X communications. To provide a secure group communication in V2X, it is necessary to create, manage, and distribute the group keys securely with a low communication overhead [2,3].

Group key management algorithms can be categorized into two types: centralized and distributed group key management. While the centralized group key management is suitable for symmetric cryptography algorithms, it suffers from high overhead in computation, communication, and storage. The distributed group key management allows every node in the group to participate in the interactive computation of the group key. Hence, it distributes the key management load to all the group members, thereby providing a higher security level and fault-tolerance in integrity and confidentiality. In view of these benefits, we chose the distributed approach [4].

Distributed key management methods are built without a central entity or authority. In these methods, each member of the group is equally trusted and required to participate in managing the keying material. Distributed key management in group communication includes the following operations: generation of cryptographic keys, exchange of keys, rekeying, and update of the keys [5]. The distributed key management methods are commonly used in ad hoc and dynamic networks.

Several solutions have proposed hybrid architectures that are built with centralized and distributed key management methods. Hybrid architectures improve the scalability and performance of group key generation and distribution processes. Therefore, the proposed architecture can also be extended to a hybrid key management approach. However, the hybrid key management approach is beyond the scope of this paper.

Moreover, confidentiality is an important security requirement for V2X communications to ensure the group’s data privacy. Confidentiality guarantees that only the vehicles within the group can access the data, while nonmember vehicles cannot understand the secured contents [1,6]. Without providing a confidentiality mechanism, group members’ messages are particularly vulnerable to different attacks in vehicular networks. In these cases, the attacker can gather information about details such as the vehicle’s location and routes, and the user’s privacy. Moreover, the attacker may cause serious problems in the future.

In current V2X implementations, several applications require message confidentiality. Only safety-related messages do not contain sensitive information. Therefore, confidentiality mechanisms are unnecessary for them. However, messages from a multitude of applications, such as infotainment content distribution, sensor data sharing and security credentials management (key and certificate management) services, toll payments, internet services over RSUs, group information sharing, and billing, use a confidentiality mechanism (e.g., message encryption method) [7]. In most of the previous approaches, message confidentiality was achieved by using the symmetric or the public key method.

Numerous academic studies have been conducted on the security and confidentiality requirements in V2X communications. Although previous studies have been able to meet most of these requirements for V2X communications, they are not fully safe. Additionally, most of them also suffer from low performance in terms of computation and communication overhead and high storage. Therefore, we propose a new encryption method for broadcast messages, which is integrated with the group key management algorithm for the confidentiality of V2X communications. We provide a detailed description and performance evaluations of the proposed method using different V2V communication test scenarios.

Our main contributions are as follows:

- First, we propose a new matrix-based Scalable and Secure Group Key Management Method for V2V group communications. It enables fast and reliable key update mechanisms to highly dynamic vehicular networks.
- Secondly, we propose a new lightweight encryption algorithm that can be easily integrated with the proposed group key management method. It provides group data confidentiality at a lower communication cost than conventional methods.
- Then, we formally analyze the security strength of the proposed method and prove that it meets the security and privacy requirements.
- Finally, we provide a performance evaluation of the proposed method using different V2V communication test scenarios.

The remainder of the article comprises the following sections. Section 2 reviews related work on group key management and the confidentiality of vehicular communication networks. Section 3 introduces the system model of the proposed group security method, while Section 4 presents a description of the proposed group key management and message encryption technique for V2V group communications is explained in Section 5. Section 6 shows the security proof and analysis of the proposed method. Section 7 analyzes the computation cost and communication overhead of the proposed method. Finally, Section 8 concludes this paper.

## 2. State of the Art

Recently, several studies have been reported in areas related to V2X communication, which include broadcasting, routing, quality of service, and security and privacy. Protecting inter-vehicular communication is of utmost importance, since malicious attacks can cause serious casualties. However, it is significantly more challenging than general wireless communications, since the messages are primarily broadcasted and vehicles are highly mobile. Several V2X security methods have been proposed and standardized by organizations such as the Institute of Electrical and Electronics Engineers (IEEE), the European Telecommunications Standards Institute (ETSI), and the 3rd Generation Partnership Project (3GPP). As most of the existing methods are designed for unicast messages, which cannot properly protect V2X broadcasted messages, new studies have been reported in the field of key management, certificate management, identity- and blockchain-based security mechanisms, as well as group encryption and digital signatures based on the public key method.

We analyze selected research with a focus on our primary concern, i.e., group key management and encryption for V2X communications.

Relatively little research has been done on secure group communication and group key management. The previous group key agreement protocols have primarily used group Diffie–Hellman, CLIQUES, tree-based group Diffie-Hellman (DH), and elliptic curve DH methods [4,5,8,9,10]. However, these protocols are not directly applicable in V2X networks because of implementation and deployment problems. The main drawback of these group key management protocols is their centralized key generation mechanism that requires key distribution from a server.

For example, Zheng et al. [11] proposed two centralized group key management protocols, Chinese remaindering group key (CRGK) and fast Chinese remaindering group key (FCRGK) based on the Chinese remainder theorem (CRT). The proposed approach is suitable for small to medium-sized dynamically changing groups, with minimized storage and broadcast messages. However, the computation cost of the CRGK was very high, especially for the key distribution operation. In 2009, Zhou and Ou [12] also designed a group key distribution algorithm based on the CRT. The main advantage of [12] is that it reduces the key server’s computation cost during group key distribution. When the number of group members increases to a certain number, the key server computing time will decrease. The algorithm of [12], however, incurs excessive costs in organizing group subtrees and computing root IDs in the group member subtrees.

In 2014, Niu [4] proposed an Elliptic curve Diffie-Hellman (ECDH)-based scalable distributed key management scheme for secure group communication. It introduced group key establishment and rekeying algorithms that depend on changes in the numbers of members. In the method of [4], distributed key generation architecture is used to reduce the key management load on the centralized server. Nevertheless, the computational cost and key size increase with the increase in group size. In addition, DH-based schemes are not an efficient solution for medium and large dynamic groups.

The protocols described above are not suitable for the rapidly changing dynamic structure of V2X networks.

In 2015, Park and Seo [8] proposed a fast group key dissemination scheme for out-of-range V2I communications. The authors considered the issue of group key dissemination for the V2V-based group communication scenario, where vehicles cannot reach RSUs for a period. However, they assume that at least one vehicle has updated its group key via a prior V2I communication. In other words, that particular vehicle had V2I communication with an RSU for group key update. The rest of the vehicles in the V2V communication group may try to update their own group keys by requesting their neighboring vehicles within the group. However, this protocol requires long request–response messages to exchange keys, which results in a significant network overhead. In addition, only the key distribution center is responsible for generating, distributing, and managing all the keys for all the vehicles. Such centralized key management systems tend to impose substantial complexity and overhead on security establishment processes. In 2016, Vijayakumar [13] proposed a dual group key management scheme for vehicular networks. In this scheme, vehicles are categorized into three classes with different authority levels to access the service. It employs CRT for the distribution and updating of a group key and thus, provides a lower computational cost than other related methods. However, key generation and management of this method require that new vehicles in the network must have a direct connection with the trusted authority (TA) for key generation and updating. This method, therefore, is not suitable for highly dynamic V2V communications that need to operate for an extended period without RSU.

In 2018, Li et al. [2] analyzed various key generation and management methods based on a secret key, group key, and subgroup keys for vehicular communications. Additionally, they suggested an effective group key management protocol that provides message authentication and confidentiality. This protocol uses a cloud-based infrastructure to solve group formation, group key generation, and key distribution issues to reduce the computational complexity. Liu et al. [14] designed a secure and efficient group key agreement scheme for vehicular ad hoc networks (VANET). They attempted to reduce the computational cost of key calculation and agreement by using powerful RSU and TA, which is similar to Li’s cloud-based infrastructure [2]. The method described in [14], however, requires vehicle registration and verification processes with TA for group key agreement. It also requires complex bilinearity for every member’s group key generation, which implies excessive computational time.

This discussion indicates that there are still many open issues in group key management in V2X communications that demand efficient approaches:

- Most of the previous methods use the V2I or TA infrastructure for the group key management process. Moreover, they did not clearly define group key generation or updates. Additionally, they did not implement a mechanism for new vehicles to join and leave the V2V network.
- In the previous methods, it is difficult and expensive to control the key update. Any change in the network structure or constraints requires the generation of a new key set. If the key update period is too long, the corresponding key may be exposed [15]. However, if it is too short, unnecessary or meaningless key updates lead to excessive overhead for the key distribution process in a highly dynamic V2X communication environment.
- Several previous methods require large key storage. For example, current V2X security standards like IEEE and ETSI require pre-stored key materials and certificates. This results in a large overhead on memory size and search time for an appropriate certificate. As storage in V2X nodes is generally limited, it is more desirable for each vehicle to generate temporary keys using only keying materials such as key generation parameters.

Attribute-based encryption (ABE) is regarded as an excellent candidate due to its ability to provide data confidentiality in a dynamic network. ABE methods can be classified as a public-key encryption method. In ABE, vehicles can share encrypted data based on attributes (e.g., vehicle ownership, vehicle types, location, and time stamp). Only those vehicles whose attributes comply with the access policy can generate a key and decrypt the encrypted data.

Huang and Verma [16] proposed one of the first ABE-based confidentiality methods for vehicular networks as a flexible and secure key management framework. This framework provides an integrated solution to data access control, key management, security policy enforcement, and secures group formation in a dynamic vehicular communication environment. In ABE methods, all usable keys and ciphertext are labeled with certain attributes that are selected based on various road conditions; therefore, the encryption keys are dynamically updated when the road conditions change.

Afterward, Liu et al. [17] proposed an extended ciphertext policy, attribute-based encryption (CP-ABE) algorithm with multiple authorities and authorized vehicles that exploit attribute-based signatures. Controlling access to network infrastructure usually requires frequent connection with the certificate authority via architectures like multi-hierarchical RSUs. In the paper by [18], the authors proposed a secure billing protocol over an attribute-based encryption method for vehicular cloud computing. A lightweight attribute-based encryption protocol is proposed to guarantee access control to the purchased services, derived from the elliptic curve integrated encryption scheme (ECIES). This protocol is used for billing transaction confidentiality for the roadside cloud zone [18]. In 2017, Xia et al. [19] proposed an adaptive forwarding scheme for multimedia data privacy preservation in vehicular networks that employ attribute-based encryption based on ciphertext policy. Moreover, the authors used the CP-ABE delegation scheme with a decision tree algorithm to forward encrypted multimedia data. Additionally, RSUs dynamically decide whether to delegate decryption of messages before forwarding them to vehicles. A decision tree offers an efficient mechanism for nodes to easily maintain network parameters such as distance measurements, the exact number of vehicles, data types, and ciphertext size. However, this scheme takes a long time to operate, as it often involves RSUs managing the key materials. A revocable access control scheme [20] using multiauthority CP-ABE to provide decryption and lower computation costs was proposed. This method can protect the network from static corruption of the authorities with low overhead of communications and computation for decryption. However, this method suffers from high computational complexity in setup, key generation, and encryption operations. In addition, its bilinear pairing is a complex and time-consuming operation. A hierarchical ABE is proposed by [21] to provide a secure message access control framework in vehicular cloud computing. This framework generates persistent or dynamic attribute keys independently for individual vehicles. Thus, it allows vehicles to share their confidential messages with other vehicles that satisfy the predefined access policy.

This review of previous work reveals that the main problem with ABE-based methods lies in the overhead of handling a large number of attributes and ensuring their independence. If to reduce the overhead, we limit the number of attributes, then the system cannot properly resist attacks. Employing more attributes increases computational overhead in the ABE data encryption and decryption phases. By using strong encryption and decryption based on persistent attributes, Ref. [21] decreases the computational overhead for access control of on-board units (OBUs) in vehicles.

Given the problems described, previous confidentiality methods are not commonly adopted for V2V communications. In summary, the key generation is controlled by the third trusted authority (RSU or TA) in all the previous methods. Furthermore, the computational complexity is unacceptably high, especially in the security initialization (setup) and in the message encryption process. Computational performance is regarded as one of the most important factors in determining the V2X security method. The V2X security overhead imposes both bandwidth utilization costs on the communication links and computational costs on the nodes. The computational cost depends on the underlying security algorithms and their associated parameters. Hence, the new framework must provide an integrated solution that can substantially reduce the computational cost in data access control, key management, security policy enforcement, and secure group formation, even under highly dynamic vehicular network environments.

In our paper, we propose a new group key management and group encryption algorithm that provides high speed and short latency. Due to the real-time behavior of V2X communication, long delays of messages can have a significant impact on V2X security applications. Therefore, this paper proposes a fast encryption and decryption algorithm for V2X networks that is used for key generation, secure certificate distribution, and secure multimedia confidential data communication among the vehicles and infrastructure. The paper covers only V2V group communication scenarios.

The proposed scheme introduces a matrix-based scalable and secure group key management method (2SGKM) and the message encryption (ME) method for V2V group communications. It enables a fast and reliable key update, as well as group data confidentiality at a lower communication cost than conventional methods.

## 3. Problem and Objectives

In this section, we discuss the problem background, introduce the system model and assumptions, and identify our design goals.

### 3.1. Background

The ITS communication security standards for V2X have been developed by dedicated working groups within the standardization organizations such as the ETSI TC ITS WG5 working group in Europe and the IEEE 1609.2 working group in the USA. The IEEE 1609 DSRC WG has developed a standard for secure wireless communications for safety applications and Wireless Access in Vehicular Environments (WAVE) management messages (IEEE 1609.2). In IEEE 1609.2, the service for message authenticity and integrity verification is based on digital signatures using the elliptic curve digital signature algorithm (ECDSA) [22].

The IEEE 1609.2 standard defines the security data structures and secure message formats. It also describes the process of secure messages within the DSRC/WAVE system [22,23]. The existing V2X security frameworks are derived from general wireless networks and, thus, have critical drawbacks as listed below:

1. *Certificate distribution*: The IEEE 1609.2 design allows a device to protect privacy by changing its certificate. The current standard uses peer-to-peer certificate distribution (P2PCD). However, P2PCD is not applicable to the group- or cluster-based communications with high dynamics and decentralized distribution. Notably, V2X communications are mainly conducted in a decentralized and ad hoc manner.
2. *Cryptomaterial management*: Previous security frameworks usually have overly long certificates that are often longer than the message. This tends to cause a substantial performance loss.
3. *Key management*: They use inefficient protocols for key generation, key lifecycle management, and key distribution, which have a large impact on secure communication performance.

**Figure 1:**
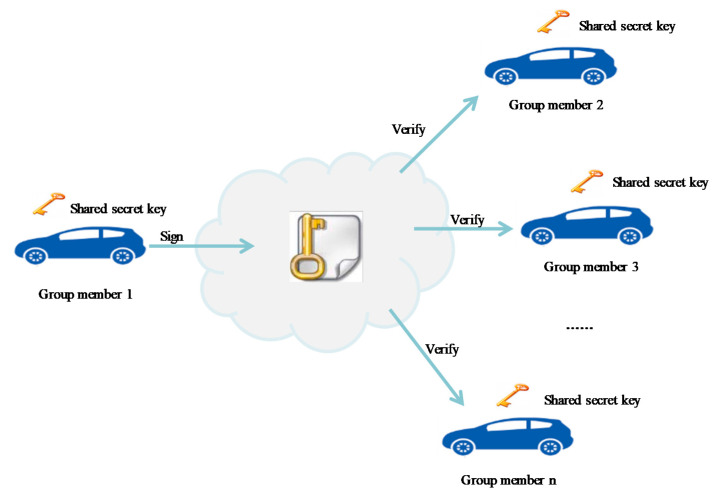
Conventional shared key management method.

IEEE 1602.2 describes the basic key management standard for secure WAVE services [23]. Most of the conventional methods use a shared secret key that must be exchanged by all the nodes for data integrity verification and confidentiality in V2X communications. Although the shared key method can be used for broadcasting V2X messages, it requires that all vehicles exchange a common key, which can expose such methods to dangers of hacking. Figure 1 illustrates such a shared key security protocol for V2X.

**Figure 2:**
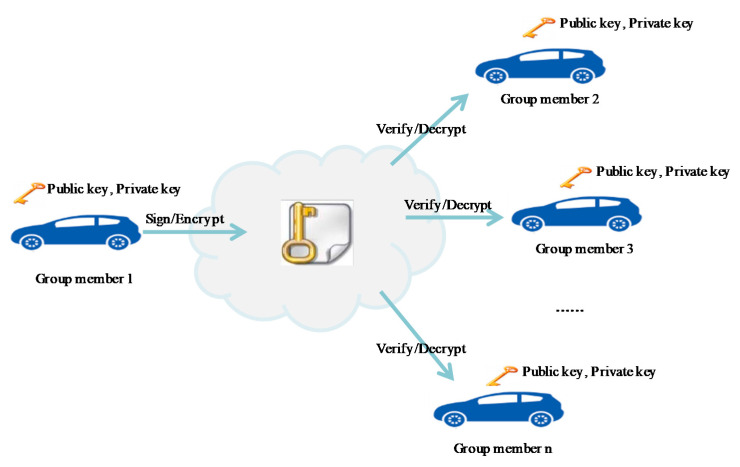
Conventional basic key management method based on the public cryptography.

Another popular conventional method is public-key cryptography, which uses a pair of public and private keys for each node in the network [22]. The main drawback of this method is that the single public and private key is broadcasted to all members or distributed with peer-to-peer communication between the members with certificates. If a hacker compromises the private key or a certificate, the hacker can listen to the communications and react to the movements of another member (Figure 2).

With conventional security protocols, it is extremely difficult to guarantee that public and private keys are used only by group members and to prevent other malicious nodes from posing as members. It is also very difficult to determine how many members are using these keys to securely broadcast data in the network. Additionally, it is highly challenging to update the keys in conventional public key-based methods.

Our objective is to allow secure and confidential broadcast communications among network members. To explain our proposal, here we define the network of nodes within a one-hop neighbor as a group. We also define the nodes within a group as group members. One of the group members is elected as the group manager, who generates the group key and distributes it to the members

### 3.2. Proposed Model

**Figure 3:**
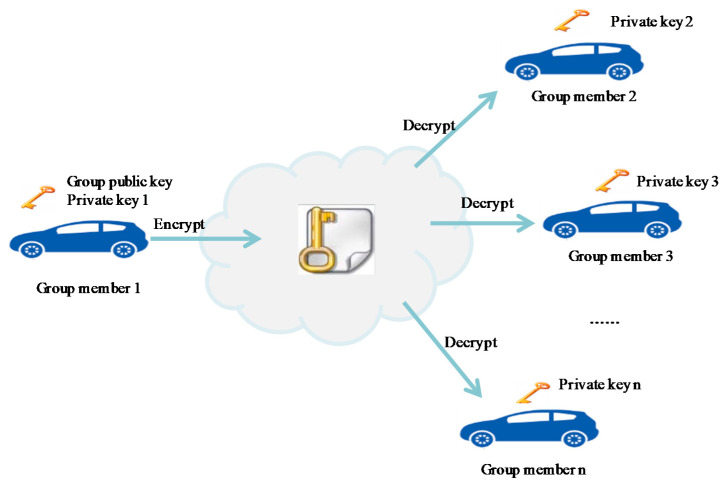
The proposed key management method for secure V2X group communication.

To address the problems listed above, we propose a group key-based public cryptographic algorithm for V2V broadcast communication. The main idea of the proposed algorithm lies in the use of a single public key for multiple private keys for each member of the group. Figure 3 illustrates an example of a group of n members, where a single group public key can be used for n private keys for all n members.

Our proposed 2SGKM provides fast asymmetric key cryptography that maintains a group public key and multiple private keys for each V2X network group. In each group, a group key is created and distributed by a group leader, thus providing efficiency in key distribution and maintenance. Groups are geodynamically formed within the leader transmission range, and keys are generated and updated based on the number of group members [24].

The properties of the proposed algorithm are summarized as follows:

- A single public key and multiple private keys are generated to support future data confidentiality and privacy in V2X group communication.
- The group manager is only responsible for the generation and distribution of group public keys.
- Each member generates its own private key; thus, private keys are never transmitted.
- The same group public key is used by all member vehicles to encrypt messages.
- Each vehicle uses its individual private key for decrypting received messages.
- If a new member joins the group, other members maintain their private keys, while only the group public key is regenerated by the group manager’s vehicle. This reduces the computation and communication overhead.
- The proposed method provides a faster computation time than the existing methods.

### 3.3. Problem Statement and Design Objectives

We know that for secure V2X communications, both security and privacy are essential. These issues are mainly solved with cryptographic operations. Cryptographic operations also place a large computational burden on receivers that wish to verify or decrypt these messages. According to IEEE 1609, a vehicle sends a basic safety message (BSM) within the time interval of 100–300 ms. Encryption and decryption of a message every 100 ms may not seem to cause significant overhead, even for the conventional public key-based schemes. However, in the case where the network has 50–200 vehicles within the communication range, each receiver needs to verify around 200–1000 messages per second, which often entails excessive computational overhead for the vehicle’s integrated system. Moreover, public key certificates have to be frequently verified as well. However, this operation is only used to provide message authentication and integrity. The integrity protection is not the focus of this study.

The requirements for network security are growing intensively with the development of new technologies and the deployment of more demanding applications to V2X communications. Signing and verifying messages are certainly not able to achieve fully secure communication. Confidentiality and reliability of messages and network members play an important role in safe driving today. Therefore, more scenarios such as the security credentials updating or multimedia contents distribution require message confidentiality, which is achieved with encryption methods. To our best knowledge, all currently available algorithms for public key infrastructure-based group communication schemes are far from satisfactory to this stringent time requirement. Moreover, cryptographic operations make the security protocol not scalable to traffic density. Therefore, the proposed cryptographic algorithms must be very fast so that the incoming messages can be processed and adapted to network dynamics [25].

Hence, we propose a new scheme for key management and data exchange confidentiality (2SGKM and ME) in V2V communications that satisfies the following security and design requirements:

1. *Secure key generation*: Key generation for V2X group member vehicles is accomplished by the group manager vehicle. All shared key materials are encrypted with the initial group key in the communication.
2. *Broadcast message encryption and decryption*: Only legitimate entities can observe the contents of a communication. Any member in the V2X network group can decrypt each broadcast message encrypted by any member.
3. *Identity privacy preserving:* The identity of the vehicle must be anonymous for any participant in communications. Any third party cannot obtain vehicle’s true identity through the message from a given vehicle.
4. *Traceability of malicious vehicles*: Only the group manager vehicle can identify the identity of the malicious vehicle. The malicious vehicle can steal other vehicles’ group PID-data table contents, but cannot join to a group communication without the correct group membership.
5. *Attack resistance*: The proposed secure group communication scheme can resist many common attacks, such as the replay attack and stolen table attack.
6. *Low communication and computational overhead*: The security protocol for V2X networks must ensure real-time key management and encryption/decryption of many messages in a short interval.

### 3.4. Lattice-Based Cryptography

In this paper, we propose a scalable and secure group key management method for V2V communication. This method employs a high-speed matrix-based encryption algorithm that is classified as a lattice-based cryptographic construction.

It is known that lattice-based constructions have strong security proofs and their implementation is relatively efficient. The security of lattice-based cryptography is based on the hard problems on point lattices in m-dimensional Euclidean space *R^m^*.

The following describes lattice-based cryptosystems in m-dimensional Euclidean space *R^m^*.

**Definition** **1.** *A lattice is a set of points in m-dimensional space with a periodic structure. For example, given n linearly independent vectors b_1_, b_2_, b_n_ ∈ R^m^ as basis vector, a lattice L in m-dimensional Euclidean space R^m^ generated by them is a set defined by Equation (1)* [26].

(1) $$L\;(b_{1},\;b_{2},\;b_{n})=\left\{\;\sum_{i=1}^{n}a_{i}b_{i}:\;a_{i}\in Z\;\right\}.$$

Here, *n* and *m* integers are called the rank and dimension of the lattice, respectively.

**Definition** **2.** *Lattice computational problems consist of the shortest vector problem (SVP) and closest vector problem (CVP), which are two fundamental challenges on the lattices.*

SVP, one of the most important lattice-based computational problems, requires approximating the minimal Euclidean length of a nonzero lattice vector [26].

**Definition** **3.** *(Shortest vector problem (SVP)) Given any basis matrix B ∈ Z^m×n^ of a lattice L (**B**), find a shortest nonzero vector **b** ∈ **L** such that || b || = D_min_ (L).*

An integer lattice ***L*** which satisfies $Z_{q}^{n}$ ⊆ *L* ⊆ *Z*^n^ for some integer *q* is called *q*-ary lattice where *q* is an integer modulus. There are two hard problems, a small integer solution (SIS) and inhomogeneous small integer solution (ISIS), related to *q*-ary lattices. The ISIS problem is more complex; solving this problem for a small solution is sufficiently difficult [27].

**Definition** **4.** *(Inhomogeneous small integer solution (ISIS)) Given an m × n integral matrix A ∈*$Z_{q}^{m\times n}$$Z_{q}^{m\times n}$*with integer modulo q*, *a real constant β and a random vector y∈*$Z_{q}^{m}$*find a vector x ∈ Z^n^\{0} such that Ax = y (mod q) and || x || < β* [27].

These ISIS problems have a number of solutions for their appropriate equations. Finding as a small solution, however, is as difficult as the worst-case problems in lattices.

These problems are regarded as extremely hard to solve in reasonable computation time, even with approximation factors that are polynomial in *n*, and even with a quantum computer. Therefore, lattice-based cryptographic constructions provide a promising future for the postquantum cryptography [26]. Since the proposed matrix-based method falls into a class of lattice-based cryptography, it can provide high level of security.

## 4. Proposed Group Key Management Method

The main objective of the proposed solution is to develop a matrix-based scalable distributed group key management scheme for V2V group communication that works with a single group public key and multiple private keys for all vehicles. In the following subsections, we describe the main elements of the proposed algorithm: V2V group organization, key generation and distribution, and key updates.

This paper focuses on V2V group communication. It is assumed that every vehicle is equipped with an OBU device. Additionally, each vehicle’s OBU contains a security module that is built based on the proposed scheme and its information never disclosed. The notations used in this paper are listed in Table 1.

Each vehicle maintains a pseudo identity data (PID-data) table in its security module that contains a set of PIDs and key generation and encryption algorithm parameters, as shown in Table 2. Vehicles always update their tables from trusted authority (TA) once they get into the radio range of an RSU. To protect the privacy, it is necessary that vehicles do not have unique pseudo identity. They use one of the pseudo identities stored in the PID-data table.

In the proposed method, the real identity of the vehicles is hidden and not used in V2V group communication.

### 4.1. Group Establishment and Group Manager Election

A group of vehicles is established by selecting vehicles moving in the same direction within the transmission range. There are various group establishment methods. Hence, we assume that one of the existing methods can be applied to establish a group.

Once a group is established, a group manager vehicle is elected, which is responsible for the generation and distribution of the public and private keys to be used in the group. For the group manager election, the existing clustering algorithms can also be used, which elects a vehicle as a group manager (cluster head) among the group members *m_i_* [28,29,30,31]. In addition, a vehicle regarded as most stable and trustable is elected as a group manager by the election algorithm. However, a complete study of the group manager election is beyond the scope of this paper.

**Figure 4:**
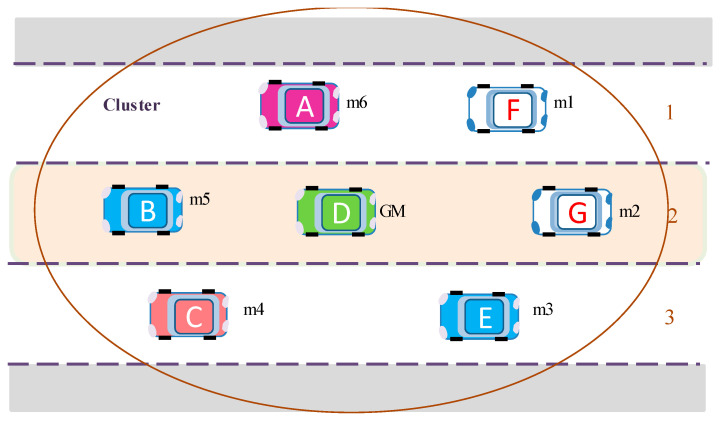
Construct cluster with group manager (GM) and members $\;m_{i}$.

Figure 4 shows an example of group establishment, where a group (cluster) is constructed and vehicle D is elected as a group manager (GM).

### 4.2. Group Key Generation

All the vehicles in the group are operating the proposed cryptosystems. The individual vehicles generate a different ternary seed matrix *A_S_* defined by Equation (2) as an *n* × *n* size ternary matrix whose elements *a_nn_* are randomly chosen.

(2) $$A_{Si}\left(m_{i}\right)\;=\;\left(\begin{array}{ccc} \begin{array}{c} a_{11}\\ a_{12} \end{array} & \cdots & \begin{array}{c} a_{n1}\\ a_{n1} \end{array}\\ \vdots & \ddots & \vdots \\ a_{1n} & \cdots & a_{nn} \end{array}\right)$$

where ${\left[As\right]}^{-1}$ (mod *p*) ≠ 0 with a small prime number *p*. The elements *a_nn_* of matrix *A_S_* are ternary values randomly chosen from [−1,0,1]. The variable *p* is chosen to satisfy gcd (*p*, *q*) = 1, and to reduce the complexity of the function by module *p*.

The group manager (GM) maintains the information of all the members (*PID_i_*, member’s matrix key data for group key generation, location (joined), and the request time to join the group) by periodically exchanging vehicle information messages such as a basic safety message (BSM) of IEEE 1609. *PID_i_* is a randomly selected unique 8-byte identity number in the PID-data table that is generated by the TA or the service provider in vehicle registration [32,33]. The member’s matrix key data for group key generation *D_i_* is computed as follows:

(3) $$D=\;{(A_{Si}\left(m_{i})\right)}^{-1}\;(\mathrm{mod}\;q)$$

Based on the matrix key data of all members, GM generates a group public key G_pub_ for broadcast communication in the group.

**Figure 5:**
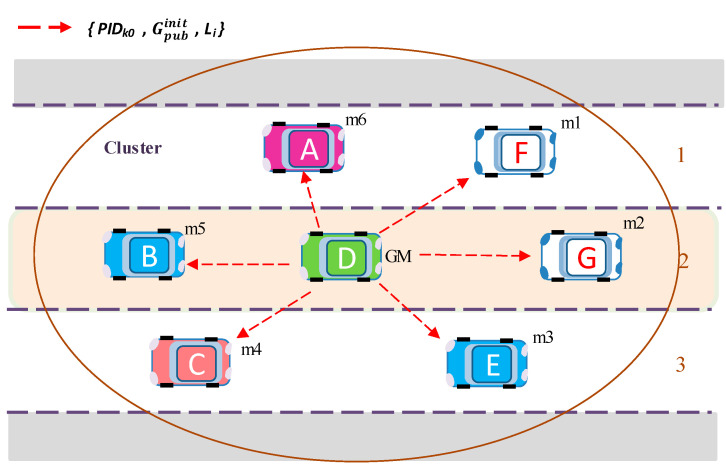
GM initial public key broadcasting.

Figure 5 illustrates the initialization step of the proposed group key algorithm. Suppose the group comprises six members, *m*_1_, *m*_2_, *m*_3_, *m*_4_, *m*_5_, *m*_6_, and vehicle D is elected as GM. Each vehicle generates its own $\;A_{Si}$ seed matrix, which is used for generating public and private group keys.

**Figure 6:**
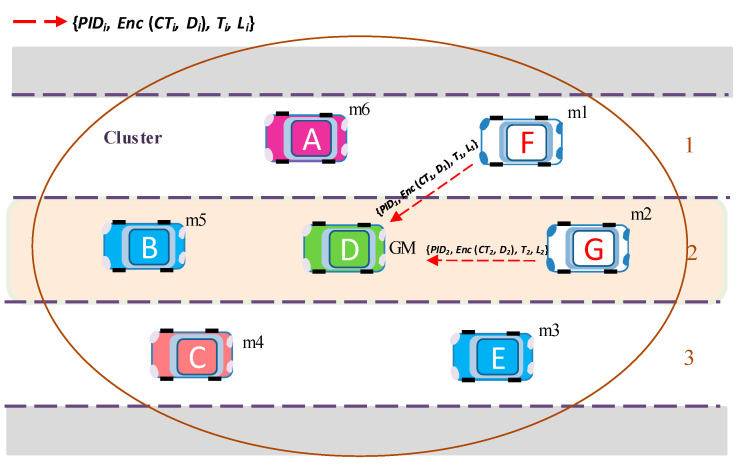
The key request messaging to GM.

**Figure 7:**
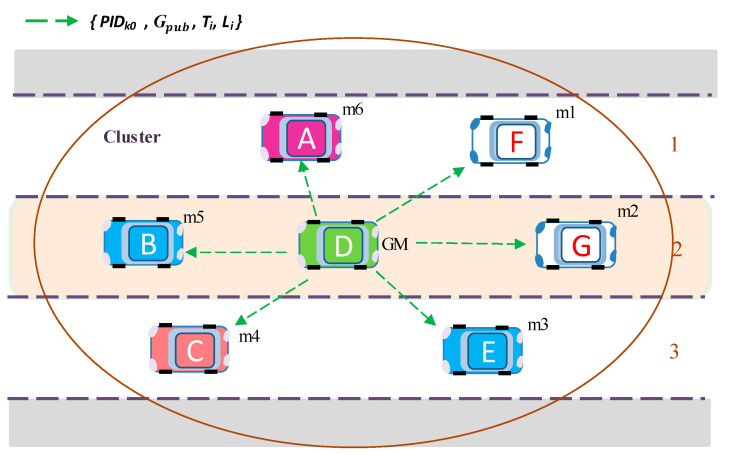
The GM broadcasting group public key *G_pub_* for group.

The key generation for the group is conducted by the following process:

- GM generates the initial group public key $G_{pub}^{init}$ and broadcasts to all members of the group with *PID_k_*_0_ (Figure 5). This key is then used by group members to encrypt key request materials. GM’s initial group public key is calculated with Equation (4):
  (4) $$G_{pub}^{init}=(p\times D_{\mathrm{GM}}\;\times \;g)\;(\mathrm{mod}\;q).$$
  Here, *D_GM_* denotes the GM’s matrix key data for group key generation. In addition, *g* is a randomly generated ternary matrix of the same size as matrix $\;A_{Si}$. The matrix *g* is generated by each GM for its group and is used to distinguish the groups.
- After receiving the initial group public key $G_{pub}^{init}$ and *PID_k_*_0_, group members configure security parameters with *PID_k_*_0_ from PID-data table, and start generating key request messages *M_i_ =* {*PID_i_*, *Enc* (*CT_i_*, *D_i_*), *T_i_*, *L_i_*} using this key. Here, *CT_i_* is contents type. The contents type *CT* is attached to the message by the sender, before encryption, and used to check the application type by the receiver after decryption.
- For example, member *m*_1_ generates its own ternary seed matrix $A_{S1}$($m_{1}$), computes *D*_1_, and makes its own key request message with encrypted contents expressed by *M*_1_ = {*PID*_1_, *Enc* (*CT*_1_, *D*_1_), *T*_1_, *L*_1_} and sends it to GM. *PID_i_* is randomly selected from *PID*_1_, *PID*_2_, *PID_k_*. The message encryption and decryption algorithms are explained in Section 5.
- Similarly, member *m*_2_ generates key request message *M*_2_ = {*PID*_2_, *Enc* (*CT*_2_, *D*_2_), *T*_2__,_ *L*_2_} encrypted with the initial group key $\;G_{pub}^{init}$, and then *m*_2_ sends *M_2_* to GM.
- All other *m_i_* members near group manager GM generate *M_i_* and send the key request messages to GM (Figure 6).
- After receiving key request messages from all members who intend to join the group, GM checks the *PIDs*, decrypts the messages and creates the information table for all members. GM saves the Media Access Control (MAC) address of a newly joining member and verifies whether the new member is a legitimate one with correct *PID*_i_. Based on the information table, GM in a V2V environment can stop a malicious vehicle from joining or rejoining the group. Afterward, the GM vehicle calculates the group public key *G_pub_* using the information for secure group communication.
- The GM vehicle uses Equation (5) to calculate the group public key *G_pub_*:
  (5) $$G_{\mathrm{pub}}=(p\times D_{\mathrm{GM}}\;\times \prod_{i=1}^{k}D_{i}\;\times \;g)\;(\mathrm{mod}\;q).$$
  Here, *k* indicates the number of members, while *D_GM_* and *g* are defined in Equation (4).
- Subsequently, GM generates a key response message, encrypted with the new group public key *G_pub_* and broadcasts it to all members with an encrypted information message (Figure 7). This message contains the *PID_i_* and the new group public key with the data contents.

Each vehicle generates its own private key *Prm_i_* using the ternary seed matrix $A_{Si}$($m_{i}$), which is used to decrypt the received messages. The private key is calculated by Equation (6), which takes the inverse of the ternary seed matrix followed by a modular operation with *p*, which is a small finite field prime integer.

(6) $$G_{\mathrm{pub}}(p\times D_{\mathrm{GM}}\;\times \prod_{i=1}^{k}D_{i}\;\times \;g)\;(\mathrm{mod}\;q).$$

Here, we calculate $A_{Sip}$($m_{i}$) as follows: $A_{Sip}$($m_{i}$) = ($A_{Si}$($m_{i}$))^−1^ (mod *p*).

Members use their private keys to decrypt the received messages, while each member uses the group public key *G_pub_* to encrypt their transmitted message.

### 4.3. Rekeying Mechanism

To provide back-and-forth secrecy in group communication, the proposed scheme must be scalable to network changes. Any change in group membership invokes the rekeying process of the group public key while satisfying the requirement that the previous group public key is still active for existing members. Contemporary research suggests that rekeying should be performed in three cases:

- When a member wants to change its key material to protect its privacy and credential;
- When a new member joins the group;
- When a member leaves the group.

#### 4.3.1. Group Rekeying Mechanism

**Figure 8:**
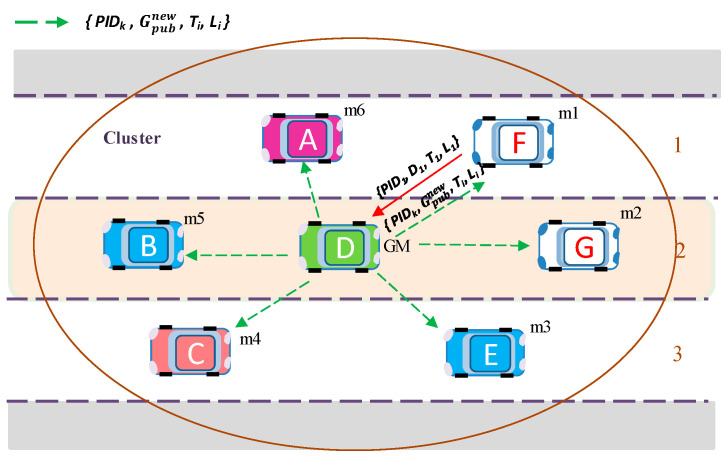
The GM updating a group public key *G_pub_* for group.

If the *i*-th member vehicle wants to change its key material, it sends an encrypted key request message *M_i_ =* {*PID_i_*, *Enc* (*CT_i_*, *D_i_*), *T_i_*, *L_i_*} to the GM with new matrix key data for group key generation *D_i_* and an appropriate *PID_i_*. Afterward, GM generates a new group public key $G_{pub}^{new}$ using Equation (5) and broadcasts $G_{pub}^{new}$ to the group in an encrypted message. Upon receiving a new group public key $\;G_{pub}^{new}$, the group members update their public key for future message encryption. For example, in Figure 8, (previously joined) existing member *m*_1_ sends a new matrix key data *D_1_*, and then GM updates the group public key *G_pub_* by a new group public key $\;G_{pub}^{new}$.

The process of updating a new group public key only requires two message exchanges with fast computation of a new group key, whenever any member (or group manager) wants to change their key materials. In addition, the updated group public key is broadcast to all members at once. Therefore, the rekeying process involves little overhead in processing and transmission time.

#### 4.3.2. Mechanism for Joining Members

When a new member joins the group, the group public key must be updated to provide backward-compatible security processing. Without a rekeying process, new members cannot access the secure group communications and, thus, cannot receive any data from the group.

Assuming that *k* members have joined the group, for secure group communication, every message carries group public key *G_pub_*. Nongroup vehicles that are near a group vehicle can also receive messages but cannot decrypt the secret contents. However, they may be allowed to extract the location of the vehicle and the public key from the message group. Suppose that a new member *m_k_*_+1_ wants to join the group. As described above, *m_k_*_+ 1_ generates a key request message encrypted with a group public key and sends it to GM. The GM generates a new group public key $G_{pub}^{new}$ with Equation (7):

(7) $$G_{pub}^{new}(p\times D_{\mathrm{GM}}\;\times \prod_{i=1}^{k+1}D_{i}\;\times \;g)\;(\mathrm{mod}\;q).$$

Afterward, GM generates a new key response message that is encrypted with $\;G_{pub}^{new}$, and GM then broadcasts to the group the encrypted information message. The information message consists of the *PID*_k + 1_, new updated group public key $\;G_{pub}^{new}$, and the encrypted data contents.

**Figure 9:**
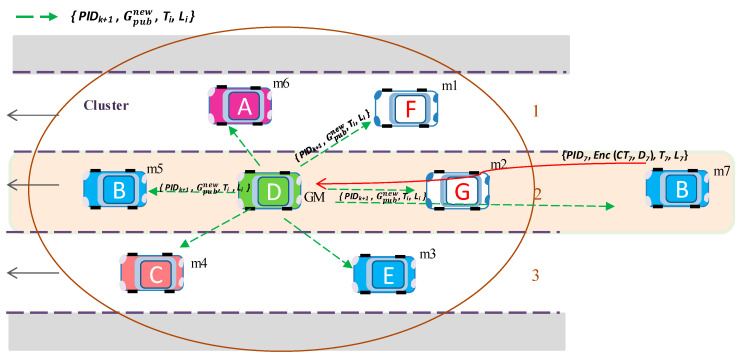
The rekeying mechanism for a new member joining the group.

Figure 9 illustrates how a new member joins the group and updates the group key. First, member *m*_7_ sends new matrix key data *D*_7_ to join the group. Afterward, GM generates a new group public key $G_{pub}^{new}$ and broadcasts it to the group.

Some group members may not receive $G_{pub}^{new}$ due to collisions or channel fading. In such cases, they can continue using the old group public key *G_pub_*. Such members using the old key, as well as members using the new key, can decrypt the messages in the group. Furthermore, all old members can also decrypt messages from new members encrypting messages using the new key. However, when some members encrypt the message using the old key, the new member cannot decrypt this message.

As described above, existing group members can also use old and new public keys for message encryption after member joining. When a new member joins the group, the member sends matrix key data for group key generation. Moreover, this member’s matrix key data is still a group public key container. In any case, these data are saved until the member leaves the group. When another member joins, GM generates a new key with the existing and new member’s matrix key materials. In that case, the old and the new group key are applied to previously joined members.

#### 4.3.3. Mechanism for Departing Members

In the case of any vehicle leaving the group, the group public key must be updated to preserve the forwarding secrecy. In the method, a special forwarding secure encryption scheme is proposed for V2X communications. This scheme adds a group key update algorithm in a member departure case, which ensures that members can use a new group public key with previous private keys in each member’s departing period to encrypt it and thus, avoid the problems caused by key exposure. Clearly, due to the unique nature of this algorithm, the departed members would not affect the group security. A new group public key ensures that the departed member cannot decrypt messages and its matrix key data are no longer valid. After member departure, some members may not receive the new generated group public key from GM. In this case, the remaining members can continue using the old key. However, it is more efficient to update the group public key for forwarding secrecy.

In the proposed method, GM checks the existence of group members in communication. If GM discovers that any member *m_k_* is not participating in the communication, GM declares the departure of *m_k_* and deletes *m_k_* from the information table of members. Afterward, GM performs a rekeying process to update the group public key $G_{pub}^{dep}$ (key updated with departing member) as follows:

(8) $$G_{pub}^{dep}=(p\times D_{\mathrm{GM}}\;\times \prod_{i=1}^{k-1}D_{i}\;\times \;g)\;(\mathrm{mod}\;q).$$

**Figure 10:**
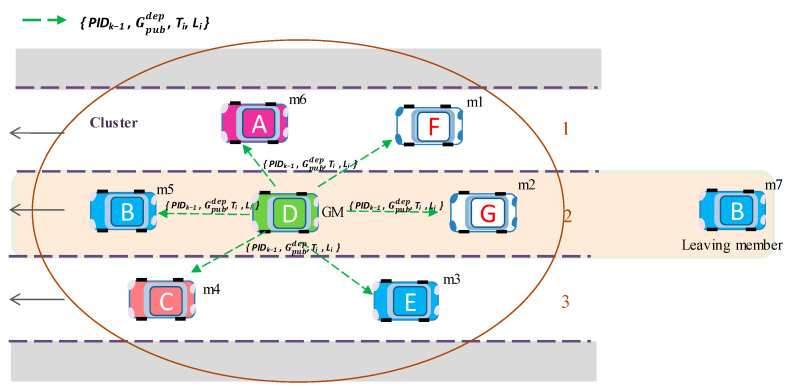
The rekeying mechanism for a member leaving.

Figure 10 illustrates an example where member *m*_7_ is leaving the group and GM generates a new group public key $G_{pub}^{dep}$ and broadcasts it to the group.

In the departure process, the private keys of existing members do not need to change. Therefore, use of the old group public key is allowed. However, members may be exposed to security attacks if the old key is used for an extended period. In this case, an intruder may compromise the old key and transmit corrupted messages.

For the members that leave the group, we do not require a special message like a departure request message. In the proposed method, we utilize a local dynamic map (LDM) updated by all the members and the group managers on the road. If GM discovers a group member moving out of its communication range in LDM, GM waits for enough departure time *T_depart_* before deleting the member. After waiting *T_depart_*, GM updates the group public key with key $\;G_{pub}^{dep}$. By using *T_depart_* in this way, we can avoid too frequent updates of the group public key.

If the GM vehicle plans to leave the group, it selects the new group manager vehicle based on the information table. We assume that a newly trusted GM receives the information table from the previous GM via a secure channel. After that, the new GM starts to control the secure group communication.

## 5. Encryption of Broadcast Messages

In this section, we explain the secure message broadcasting operation from GM to vehicles and between member vehicles in a group. To improve confidentiality, the messages should be exchanged in an encrypted form so that unauthorized or nonmember vehicles cannot access the messages. The steps involved in the secure message transmission in vehicular communications are described below.

**Message Encryption** (**ME**): In our paper, we propose a high-speed matrix-based message encryption algorithm with low latency. This algorithm is designed by using lattice-based cryptographic construction. Lattice-based cryptography is a generic term for constructions of cryptographic primitives that involve lattices, either in the construction itself or in the security proof. Lattice-based constructions are currently important candidates for postquantum cryptography. Unlike more widely used and known public-key schemes (RSA (Rivest-Shamir-Adleman), Diffie–Hellman, bilinear pairings based or elliptic-curve cryptosystems), which are easily attacked by a quantum computer, some lattice-based constructions appear to be resistant to attacks by both classical and quantum computers. Furthermore, many lattice-based constructions are more secure under the assumption that certain well-studied computational lattice problems cannot be solved efficiently.

The proposed algorithm is an asymmetric encryption algorithm supporting a single public key for the group and multiple private keys for group members. Message encryption starts by generating a group public key for the joined member vehicles.

Given a message *msg_i_* of a member *m_i_*, the encryption of *msg_i_ =* {*P*_1_, *P*_2_, *P_k_*, *P**_K_***} using group public key *G_pub_* is represented by Equation (9).

*C_k_* = (*r* × *G_pub_* + *P_k_*) (*modq*). (9)

Here, *C_i_^msg^ =* {*C*_1_, *C*_2_, *C_k_*, *C**_K_***} is the resulting cryptotext, while *r* indicates a square matrix of order *n* that is randomly generated in the interval [−1, 0, 1]. In addition, *msg_i_* is the payload for encryption of member *m_i_* with block containers {*P*_1_, *P*_2_, *P_k_*}.

**Figure 11:**
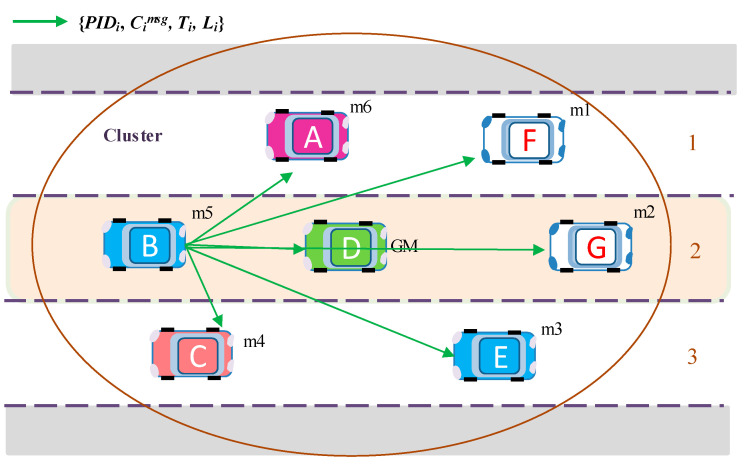
Encrypted message broadcasting in the group.

An encrypted message, supplemented with the encryption timestamp and vehicle position information *M_i_ =* {*PID_i_*, *C_i_^msg^*, *T_i_*, *L_i_*} is broadcasted to the group members, as illustrated by Figure 11.

**Message decryption**: Upon receiving encrypted messages *M_i_ =* {*PID_i_*, *C_i_*, *T_i_*, *L_i_*}, vehicles in the group decrypt the message using their private key *Prm_i_* and check its usability with the timestamp and other parameters. Only valid messages are loaded into the applications.

For any member of the group, a received cryptomessage *C_i_^msg^ =* {*C*_1_, *C*, *C_k_*, *C**_K_***} is decrypted by the decryption process as follows:

(10) $${P_{k}}^{\prime }\;=\;(((A_{Si}\left(m_{i})\;\times \;C_{k}\right)\;(mod\;q))\;(mod\;p)\;\times \;A_{Sip}(m_{i}))\;(mod\;p).$$

where *P_k_′* denotes the resulting decrypted message and *Prm_i_ =* {$A_{Si}$($m_{i}$), $A_{Sip}$($m_{i}$)} is the private key of the *i-*th member. Here, we calculate the total decrypted payload with *msg_i_′ =* {*P*_1_′, *P*_2_′, *P_k_′*, *P**_K_**′*}.

Using the encryption and decryption process based on the group key generation method described above, the proposed method can substantially improve vehicular communications security and, thus, ensure the security of both autonomous and regular vehicles.

**Figure 12:**
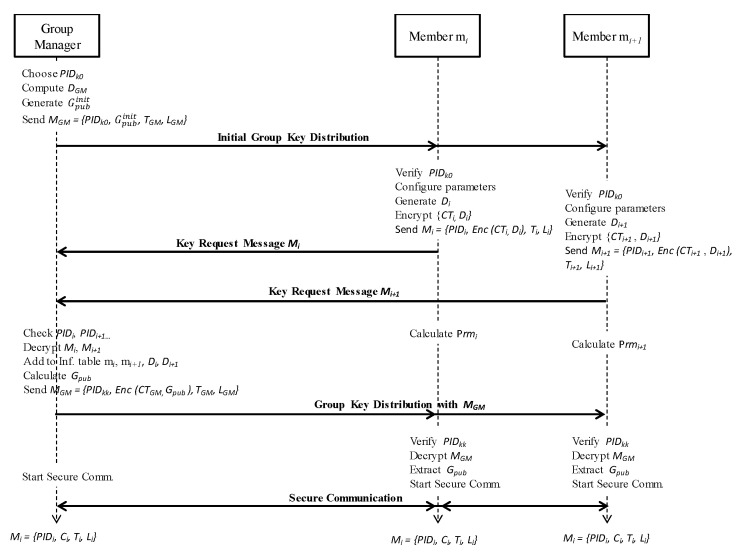
The workflow of the proposed solution.

Finally, the overall flow of message exchanges between the group manager (GM) and group members is shown in Figure 12, including the flow of message encryption and decryption.

## 6. Security Analysis

In this section, we discuss the security analysis of the proposed method for vehicular communication. We prove the security of the proposed method with formal security analysis and security requirement analysis. In order to demonstrate this, we analyze our method under a random oracle model [34] and ensure that it meets the security and privacy requirements mentioned in Section 3.3.

### 6.1. Security Proof

**Theorem** **1.** *Equations (9) and (10) used in the proposed method are correct.*

**Proof** **.** When a receiver receives a message *M_i_ =* {*PID_i_*, *C_i_*, *T_i_*, *L_i_*} from vehicles, it extracts *C_i_* {*C*_1_, *C*_2_, *C_k_*} cryptotext and decrypts it *C_k_* with its private key *Prm_i_*. If the verification key decrypts the message correctly, the algorithm is proved. □

The correctness proof of the proposed algorithm is given by Equation (10). We solve Equation (10) with Equations (3), (5), and (9) to find *P_k_′ =* *P_k_.*

(1) ($A_{Si}$($m_{i}$) *× C_k_*) (*mod q*) *=*$A_{Si}$($m_{i}$) *×* (*r × G_pub_ + P_k_*) (*mod q*) = ($A_{Si}$($m_{i}$) *× r × G_pub_ +* $A_{Si}$($m_{i}$) *×P_k_*) (*mod q*) = ($A_{Si}$($m_{i}$) *× r ×* (*p × D_GM_* ×$\overset{k}{\underset{i=1}{\prod }}D_{i}$ *×* g) (*mod q*) *+* $A_{Si}$($m_{i}$)×*P_k_*) (*mod q*)
(2) (1) (*mod p*) =*(* $A_{Si}$($m_{i}$) *×P_k_*) (*mod q*) (*mod p*) *=* ($A_{Si}$($m_{i}$) ×*P_k_*)
(3) $(A_{Si}$($m_{i}$*) ×P_k_*) *×*$A_{Sip}$($m_{i}$)) (*mod p*) = *P_k_*.

Since $A_{Si}$($m_{i}$) *×* $A_{Sip}$($m_{i}$) *=1*, *P_k_*′ *=* *P_k_* is proved. Thus, it is verified that Equations (9) and (10) are correct.

To analyze the security proof in the proposed group key generation and message encryption method, we construct a game between a challenger *C* and an adversary *A* based on the network model and the adversary’s ability. Suppose that adversary *A* tries to attack the proposed method. During the game, A can make the queries to *C*.

**Theorem** **2.** *The proposed protocol for V2V communication is secure in the random oracle model. Any polynomial adversary A cannot break the proposed method described in Section 4 with a non-negligible probability.*

**Proof.**  As the first step, the GM broadcasts a message about security initialization with *PID_k_*_0_ and $\;G_{pub}^{init}$. Suppose that there is an adversary A that can forge a message *M_i_ =* {*PID_i_*, *Enc* (*CT_i_*, *msg_i_*), *T_i_*, *L_i_*}. We can construct a challenger *C*, which could solve the short vector problem (SVP) and inhomogeneous small integer solution (ISIS) problems with a non-negligible probability leading to winning the game. Given an instance *PID_i_* and *G_pub_* of the SVP/ISIS problem, *C* simulates oracles queried by A as follows.

*Setup and Send*—*Oracle*: In this query, *C* sets the *PID_k0_* and parameters, and generates an initial group public key $G_{pub}^{init}\;$ of the group. Then, *C* broadcasts the initial group public key and *PID_k_*_0_ to the group with A.

*h1*—*Oracle*: In this query, the challenger *C* maintains information table *L* of group with the entries *m_i_* (*i* = 0, 1, 2, …), *PID_i_*, and *D_i_.* This table is initially empty. The adversary *A* generates an *h1*—*oracle* query with a key request message. Upon receiving *A*’s query, *C* checks whether the *PID_i_* exists in the PID-data table, first. If so, *C* decrypts the encrypted field of the message, extracts *D_i_* after verification of the contents type *CT_i_* with the *Decryption*—*Oracle*, and adds to the information table. Then, *C* generates new group public key *G_pub_* and broadcasts it to all members with *A*.

*h2*—*Oracle*: In this query, after *C* receives a key request message *M_i_*_+1_ = {*PID_i_*_+1_, *Enc* (*CT_i_*_+1_, *D_i_*_+1_), *T_i_*_+1_, *L_i_*_+1_} from *A*, it checks if *m_i_*_+1_(address of node) does not exist in *L*. If so, *C* checks whether the *PID_i_*_+1_ is in PID-data table. If so, *C* decrypts the encrypted field of the message, extracts *D_i_*_+1_ after the verification of the contents type *CT_i_*_+1_ with the *Decryption*—*Oracle*, and adds it to the information table as a new member *m_i_*_+1_. Then, *C* updates the previous group public key *G_pub_* with new public key $G_{pub}^{new}\;$ and broadcasts it to all members with *A*. Otherwise, if decryption or verification of the *CT_i_*_+1_ fails, *C* terminates the game. Then, *C* broadcasts a notification message about *A*.

*Decryption*—*Oracle*: In this query, the challenger *C* maintains information table *L* of group members. The adversary *A* broadcasts a message to the group. In response, after *C* receives a secure message *M_i_ =* {*PID_i_*, *C_i_*, *T_i_*, *L_i_*} from *A*, it verifies the *m_i_* and *PID_i_* and time stamp *T_i_* for freshness of the message. If they are OK, *C* decrypts the encrypted ciphertext field of the message with Equation (10). Then, *C* checks the message contents type *CT_i_*. If the contents type *CT_i_* fails, the message *M_i_* will be discarded. If above process is repeated by *A* with another *PID_i_*, then, *C* broadcasts the notification message about A to the group. □

### 6.2. Analysis of Security Requirements

We also analyze the security of the proposed method informally and show that the proposed method meets the various security and privacy requirements.

**Theorem** **3.** *The proposed method for V2V communication can effectively mitigate the potential threats, such as common attacks, malicious GM, and users’ collusion, and meet the security requirements listed in Section 3.3.*

**Proof.**  We prove our proposed method meets the security requirements shown as follows:

1. *Secure key generation*: According to the method described in Section 4.3, each vehicle generates its own private key. The private key generation details are kept only within each user and are not transmitted over communication channels, which prevents it from being intercepted or disclosed. To protect the group key generation process, the group manager generates an initial group public key $\;G_{pub}^{init}$ and broadcasts it to all members of the group with an appropriate pseudo identity. Only the legitimate group manager vehicle can generate an initial group public key with a valid pseudo identity *PID_k_*. An initial group public key $\;G_{pub}^{init}$ secures the key request and response messages from an attacker. Without a correct PID-data table contents and an initial group public key, the attacker cannot generate an appropriate private key. Therefore, secure key generation is guaranteed.
2. *Confidentiality* (*broadcast message encryption and decryption*): In order to transmit the messages securely, the group data are encrypted with a group public key and the proposed encryption algorithm. Only legal vehicles can obtain the correct decryption using its private key. Assume an attacker steals the PID-data table contents and intercepts the messages *M_i_ =* {*PID_i_*, *C_i_*, *T_i_*, *L_i_*}. However, it is difficult for the attacker to decrypt messages without owning the correct private key.
3. *Identity privacy preserving*: The proposed method provides identity privacy protection using pseudo identities {*PID*_1_, *PID*_2_, *PID_k_*}, which are linked with the key generation and encryption algorithm parameters, for V2V communication. It is not possible to identify or extract the real identity of the vehicle in V2V communications. Note that the real identity of the vehicle is disclosed only to the TA through a secure channel. Therefore, the proposed method preserves the identity privacy.
4. *Attack resistance*: We prove that our proposed method can resist many common attacks.
  - Replay and modify attacks: The timestamp Ti is included in the message. Vehicles could find the replay of the message by checking the freshness of the timestamp Ti. In addition, old replay messages are discarded by checking PIDk. In V2V communication, all contents are encrypted, and it is not possible to access or modify the contents without the private key. Any modification of the messages could be found by decryption, and an attacker cannot join the group using modification attacks. Thus, the proposed method could withstand message replay, modification, and man-in-middle attacks.
  - Impersonation attack: An attacker may try to impersonate any group member for malicious purpose, but it cannot generate a correct encrypted message. According to the Equation (10), other vehicles could detect the attack easily by checking whether the above equation holds true or the PIDi is correct. Hence, the proposed method could withstand the impersonation attack.
  - Compromised or stolen table attack: By the analysis in Section 4, the group members do not need to save the keys in a table. Instead they just need to hold initial parameters and pseudo identities only for the PID-data table, which are used for configuration and verification. The table does not store any group or private key. Then, an adversary cannot steal any verifier table from the OBU’s security module, for malicious attacks. If the attacker compromises the table, it does not provide valid decryption without a unique private key. This shows that the proposed method can resist the stolen table attack. □

In addition, there is another potential threat which may be from a malicious vehicle or GM. In the first case, the private key may be leaked by a misbehaving member vehicle to a nonmember vehicle. Nevertheless, the private key cannot be used for message decryption without an appropriate PID-data table. As we mentioned above, we assume that the security module of the OBU is tamper-proof device and its information is never disclosed. Moreover, if GM discovers that any member m*_i_* colludes with a nonmember, the GM deletes its key data *D_i_* from the group key, and m*_i_* cannot communicate with the group anymore.

In the second case, a malicious GM may control the group. Until today, there is no complete solution for the identification of misbehaving or intruder vehicles. Although the misbehaving vehicle and intruder detection is another important research area in vehicular communications, it is not our primary goal in this paper.

## 7. Simulation Results

In this section, we first evaluate the performance of our algorithms in terms of computation, complexity, communication cost, and storage requirements. Afterward, we describe the simulation setup and analyze the simulation results of the proposed algorithms. In addition, we compare the performance of the proposed method with previous works.

### 7.1. Computation Cost of the Proposed Method

In this subsection, we evaluate the performance of the proposed algorithms in terms of computational cost. Our proposed algorithms mainly include group key generation, message encryption, and decryption processes. Based on equations Equations (5), (9), and (10) in Section 4, we calculate the computation times of the proposed algorithms with the following notations (Table 3):

- Computation time of the addition of two matrices, *T_add_*;
- Computation time of the multiplication of two matrices, *T_A×A_*;
- Computation time of the multiplication matrix to number, *T_A×Num_*;
- Computation time of the modular operation, *T_mod_*.

These cryptographic operations are measured using a computer with the following specifications: Intel Core i5 (2.5 GHz) CPU, 8 GB RAM, and Ubuntu Linux 18.04 OS. In the proposed method, we used lattice-based public-key cryptography with an 8 × 8 size matrix calculation. All algorithms are implemented using C code.

We analyzed existing schemes in terms of the performance of key generation, message encryption, and decryption. Several papers [17,18,19,21,35] proposed attribute-based encryption methods developed with pairing-based cryptography (PBC). For a fair comparison, we implemented the attribute-based methods and conducted simulations using the PBC library, which supports the pairing operation [36]. A 160-bit group order elliptical curve was chosen, and the computation time measured from the simulations is shown in Table 4.

Table 5 shows the comparison results of computational costs using Table 3 and Table 4. Our method is compared with [19,21] for the following security operations: secret key generation, message encryption, and decryption conducted on the OBU, which was explained in [21].

First, we analyze the computational costs of the secret key generation phases of the methods under comparison, including the proposed method. The methods of [17,19,21,35] use an RSU over TA to generate secret keys. The methods of [19,35] perform (3 + *N_d_*) *T*_0_ operations to generate all secret keys for vehicles. On the other hand, the methods proposed by Huang et al. [21] and Liu et al. [17] show that the attribute authority (AA) only needs to generate secret keys according to dynamic attributes for vehicles because the value of the persistent attributes does not change. In these methods, however, the key generation time is two times longer than the methods proposed in [19,35]. Analyses show that the secret key generation for attribute-based cryptosystems is a long process that depends on the number of attributes. If the number of attributes or vehicles increases, the process of secret key generation also tends to grow accordingly.

In contrast, our proposed algorithm is a distributed key generation method using only OBUs. In our method, each vehicle can generate its own private (secret) decryption key at a very low computational cost. Each vehicle can communicate directly with other vehicles. Therefore, our method can still operate in areas where no RSU or infrastructure is available. Table 5 compares the key generation times among the three methods. As shown in the table, the private key generation time does not depend on the number of vehicles.

Second, we analyze the papers that use group key management methods and compare them with our proposed method. For this, we consider three performance metrics in our proposed method, namely, computation time, storage overhead, and communication cost for updating the group key for secure communication among group members. The computation time is defined as the time taken to compute the group key at the GM (or trusted authority (TA)) when group membership changes in the V2V communication group. The communication cost is defined as the delay taken to broadcast a certain amount of information from GM to provide V2V group members with an updated group key. Table 6 shows the computation time and storage overhead of various key management approaches, namely, a secure and efficient group key agreement (SEGKA) [14], fast-Chinese remainder group key (FCRGK) [11], key-tree Chinese remainder theorem (KCRT) [12], number theory research unit (NTRU) [37], Elgamal group key management (EGKM) [37], VANET group key management (VGKM) [13], and our scalable and secure group key management (2SGKM) method. The notations used for the comparison are defined as: *O* represents the computation cost in the order of magnitude of the operations, *n* is the number of group members, *τ* is the maximum number of children of each node in the tree, and *EEA* indicates the time taken to find the inverse element of a multiplicative group using the extended Euclidean algorithm. In addition, *exp* represents the exponential operation, *mod* indicates the modular operation, *Mult* is the multiplication operation, and *Div* represents the division operation. Finally, the *Add* and *Sub* represent the addition and subtraction operations.

From Table 6, it is evident that all the existing approaches imply a greater computational complexity if they are used in the GM or TA side of the VANET. This is due to their complex group key computation for join/leave operations for a single user. All existing approaches, with the exception of VGKM, require excessively higher computation costs than the proposed approach. It only takes one multiplication and one modular operation for the proposed approach to process the join/leave operation for a single user. Table 6 also compares (in the last row) the communication cost for updating the group key. Clearly, the proposed 2SGKM method provides a better or equivalent communication cost compared to other methods.

**Figure 13:**
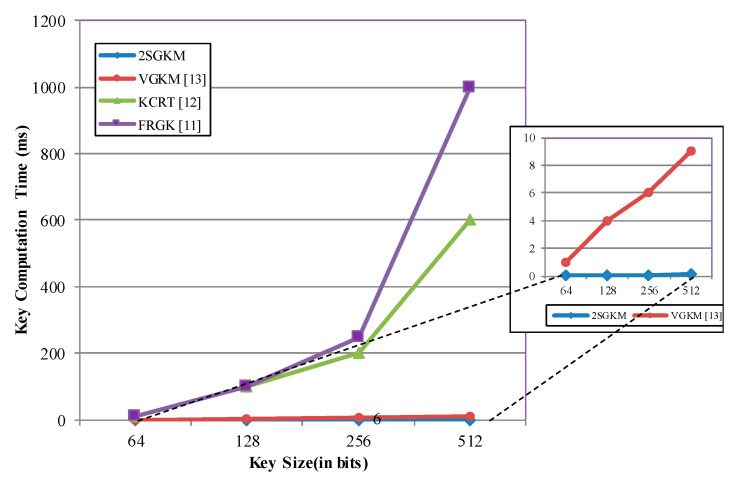
Group key generation time for different key sizes at group manager/trusted authority (GM/TA).

Figure 13 illustrates the key generation time comparison for a key length range. It shows that the proposed method 2SGKM generates almost no additional overhead in the group key generation time for large changes in key size, whereas all previous methods result in a steep increase in the key computation cost along with the increase in key size.

**Figure 14:**
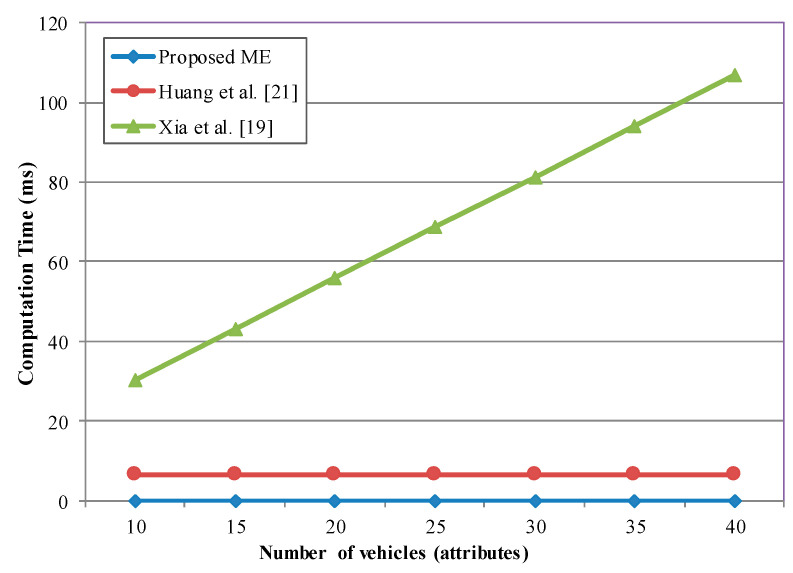
Computation cost of message encryption.

Additionally, we discuss the computational cost of message encryption and decryption. Several previous methods such as Liu et al. [17], Xia et al. [19], and Liu et al. [35] employ complex algorithms like ABE. Thus, their computational costs for vehicle-side encryption grow along with the number of attributes in the ciphertext N_c_. The proposed method not only provides constant computation time (Figure 14), it also shows a much shorter encryption time than the previous methods [17,19,21,35]. For example, the message encryption (ME) time is 100 times faster in the proposed scheme than Huang’s scheme [21] for the number of attributes N = 10.

In the message decryption process, vehicles use secret or private keys to decrypt encrypted messages. We calculated the decryption time in an OBU for the proposed method, as in the previous methods. In comparison, we need to emphasize that the methods reviewed [19,21,35] used a single pairing operation to decrypt the messages. The pairing operation is a time-consuming cryptographic operation; therefore, the computation time is high.

**Figure 15:**
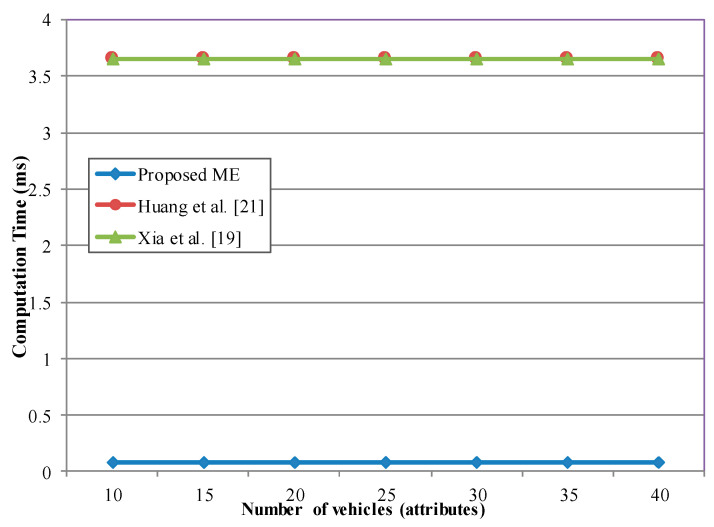
Computation cost of message decryption.

In the methods of [19,21,35] and the proposed method, the message decryption time is always constant, even if the number of the attributes or vehicles changes. However, as shown in Figure 15, the proposed method consumes significantly less computation time due to our low algorithm complexity. For example, the computation time is 400 times faster in the proposed scheme than in the schemes of Huang [21] and Xia et al. [19] for the number of attributes N = 10.

Although the proposed group encryption method is similar to Elliptic Curve Cryptography (ECC)-based methods in the use of small keys, the proposed method can provide higher performance at a lower computation cost compared to ECC (ECIES and ABE) and RSA, which is especially true for long messages.

### 7.2. Communication Cost of the Proposed Method

**Figure 16:**
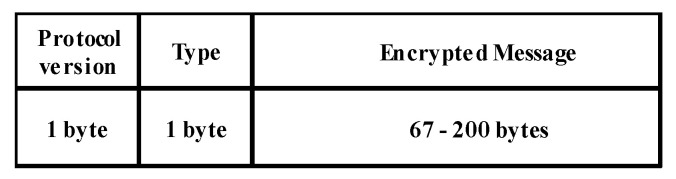
The format of an encrypted message for IEEE 1609.2.

The IEEE 1609.2 standard [22] specifies security services for vehicular communication networks. Figure 16 shows the format of an encrypted message based on IEEE 1609.2, which is explained in [22,38].

Afterward, we analyze the communication cost of various messages in our approach. The proposed method uses different formats for key generation and messaging. The message formats are adopted from [24,39].

**Figure 17:**
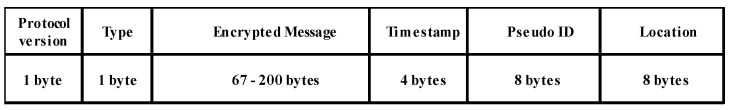
The format of an encrypted message for member vehicle.

In vehicle-to-vehicle communication, the encrypted message is generated either from vehicles that want to join the group or from those in the group. This message consists of nonencrypted fields and encrypted message field. The nonencrypted fields comprise the protocol version (1 byte), type (1 byte), pseudo identity (8 bytes), and timestamp (4 bytes). Additionally, the encrypted message field comprises the contents type (1 byte), and original message (minimum 67 bytes). Figure 17 illustrates these fields.

The contents-type field indicates whether the message is used for the key generation or for group service message exchange. Special values are used to distinguish the message types.

The communication overhead of encrypted messages of group members is only 22 bytes, indicating that the proposed method is a light-weight approach. The proposed protocol shows a communication overhead at least 30% lower than that of any previous method.

**Figure 18:**
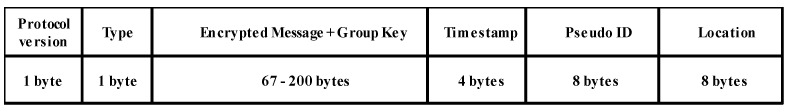
The format of an encrypted message for GM.

In GM-to-group communication, GM generates encrypted messages to the group to share secured information or update the key. An encrypted message consists of two parts: plain text fields and encrypted fields. Plain text fields comprise one byte for the protocol version, one byte for type, 4 bytes for the timestamp, 8 bytes for pseudo identity, and 8 bytes for location information. Encrypted fields include one byte for the contents type, 32 bytes for the group public key, and a minimum of 67 bytes for the original message (Figure 18).

The total size of the GM encrypted message is increased by 54 bytes, which were added by the proposed method. In summary, the proposed method can transmit encrypted messages with relatively low communication overhead. Therefore, it can provide confidentiality and authentication more effectively than conventional encryption or digital signature methods.

### 7.3. Simulation Setup and Network Performance Evaluation

Finally, we present the performance evaluation of the proposed method using network simulation. We used the NS-3 simulator, a popular network simulator supporting vehicular networks based on IEEE 802.11p, along with NetAnim, a visual mobility model for vehicle simulations. We configured a highway scenario based on an IEEE 802.11p vehicular network with a data rate of 6 Mb/s. Moreover, we configured the vehicles in the simulation with a randomly selected speed of 10 to 40 m/s (36–144 km/h). The details of the simulation parameters are shown in Table 7.

**Figure 19:**
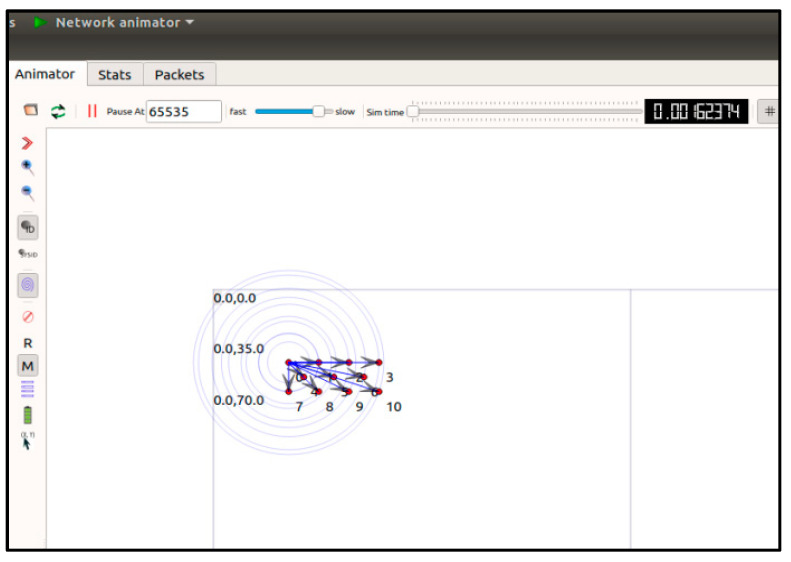
V2V secure group communication simulation in NS-3.

As described in Section 4, we implemented the proposed algorithm in NS-3 and measured its communication performance under V2V network scenarios. Figure 19 shows a network comprising a group of 11 vehicles, consisting of ten member-nodes and one GM node. In the simulations, vehicles equipped with IEEE 802.11p radios communicate over an idle channel. Figure 19 illustrates an example simulation constructed by NetAnim + NS-3 simulation tools.

The performance of the proposed scheme was measured in terms of the network characteristics and the average message delay. Additionally, the message delay measurement was conducted in relation to the group broadcast scenario. First was by the number of vehicles and second, by the velocity of the vehicles.

#### Average End-To-End Delay

Average end-to-end delay is defined as the time it takes to transmit a message from a source to a destination. The average end-to-end delay can be obtained by averaging the end-to-end delay of all the successfully delivered messages. The average end-to-end message delay includes all the possible delays in the network, i.e., buffering latency, transmission time, processing delays in the MAC operation, and propagation delay.

The average message delay (MD) is calculated by Equation (11) based on the formula defined in [3,33]:

(11) $$MD=\frac{1}{N}\;\sum_{i=1}^{N}\frac{1}{MK}\;\sum_{m=1}^{M}\;\sum_{k=1}^{K}\;\left(T_{recv}^{i,k,m}-\;T_{send}^{i,k,m}\right).$$

where *N* is the number of vehicles, *M* is the number of messages sent by *vehicle_i_*, *K* indicates the number of adjacent vehicles within the DSRC communication range of *vehicle_i_*. $T_{recv}^{i,k,m}$ represents the moment *vehicle_k_* receives the *m*-th message from *vehicle_i_* in the application layer, while $T_{send}^{i,k,m}$ represents the moment *vehicle_i_* sends the *m*-th message to *vehicle_k_*.

**Figure 20:**
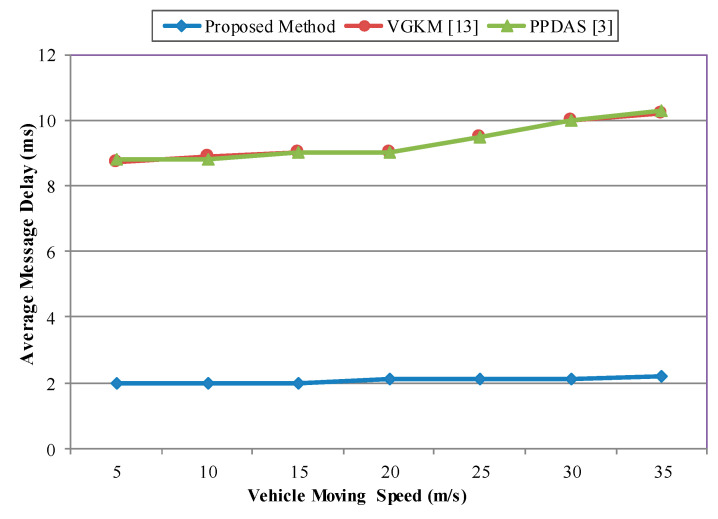
Average message delay vs. vehicle speed.

**Figure 21:**
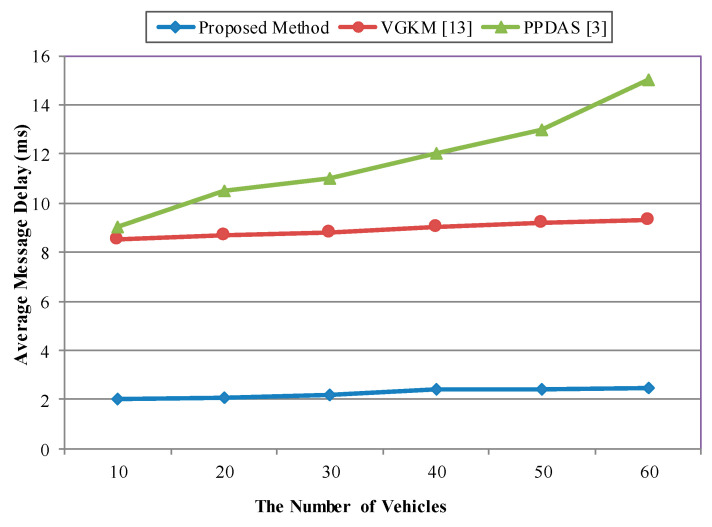
Average message delay vs. number of vehicles.

**Figure 22:**
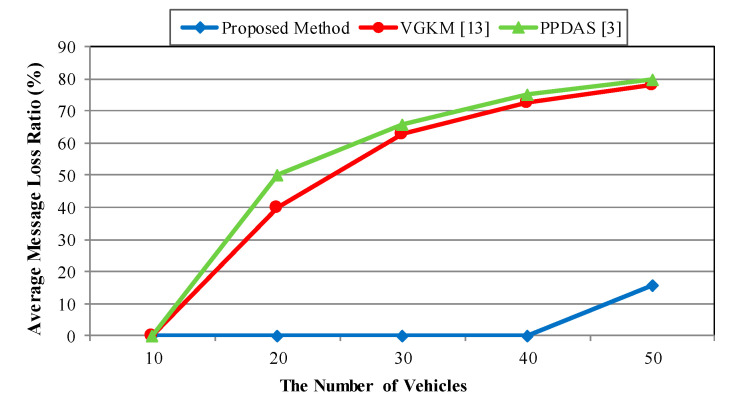
Average message loss ratio vs. number of vehicles.

We conducted simulation measurements for the proposed group key and encryption method and compared the performance with the four previous methods [3,13,19,21]. We then analyzed the influence of the vehicle density and vehicle moving speed on the average message delay (MD) and average message loss ratio (LR). The comparison results are shown in Figure 20, Figure 21 and Figure 22. Figures illustrate only the methods with low average MD ([3,13] and the proposed method). The results show that the proposed method increases network performance by 70–80% compared to previous schemes.

Figure 20 shows the relationship between MD and vehicle speed, where the x-axis represents the average speed changing from 10–40 m/s (36–144 km/h). As shown in this figure, MD in the case of VGKM [13] and PPDAS [3] increases along with speed when the speed of vehicles is higher than 40 m/s. In contrast, the proposed method delivers a nearly constant MD value regardless of speed changes, while offering a message delay that is five times shorter.

Then, we analyzed the influence of the vehicle density on the average MD. The number of vehicles varies from 10 to 60, and the average speed of vehicles is approximately 20 m/s in the experiments. The relationship between the MD and the number of vehicles is shown in Figure 21. While the average MD of VGKM [13] increases slightly with increasing number of vehicles, the average MD values for PPDAS [3] increases considerably along with the number of vehicles.

The average MD of the proposed method remains at a nearly constant value for a wide range of the number of vehicles in the network.

The average message loss ratio (LR) refers to the ratio of the number of messages dropped to the total number of messages received in each vehicle. Here, we assume that the message loss occurs by the security protocol overhead and the buffer space overflow in the vehicle, rather than the bit errors from wireless transmission channel.

Figure 22 describes the relationship between the average LR and the number of vehicles. For the case of the previous work, VGKM [13] and PPDAS [3], when the number of vehicles is larger than 11, the average LR rapidly increases along with the number of vehicles and the average LR reaches 70% when the number of vehicles is 40. In contrast, for the case of the proposed method, the average LR remains nearly 0 until the vehicle density grows to 42 vehicles.

From these simulation results, it is evident that the proposed method improves the level of security and the confidentiality of group communication between vehicles. Furthermore, it can accelerate the public/private key generation process, as well as data encryption in the network, with substantially lower message delays compared to conventional methods.

## 8. Conclusions

We proposed a scalable and secure distributed group key management method with a message encryption algorithm for V2V broadcast group communication. The proposed method provides the group vehicular communication security using a common group public key for the group with different private keys of group members. In the implementation of the proposed method, the group manager vehicle must be selected for generating group public keys by using clustering algorithms. All vehicles use the group public key to encrypt messages, and each vehicle uses its own private key for decrypting received messages. The proposed method satisfies all security requirements for key management and message confidentiality. Formal security algorithm analysis and extensive simulation results show the enhanced performance and effectiveness of the proposed method for V2V group communication. Therefore, the proposed method is applicable for both small-scale and large-scale V2V group communications.

In a future work, we will investigate the proposed method under different application scenarios of V2X communications. In this regard, we will further evaluate group key management issues with overlapping clusters and LTE/5G infrastructure.

## Figures and Tables

**Table 1:** Notations and descriptions.

Notation	Description
*p*	A small prime integer of finite field
*q*	A large prime integer of finite field
*m_i_*	The *i*-th group member; *i* ∈ [1, n]
*n*	The order of square matrix
*A_S_*	The *n* × *n* size ternary seed matrix [−1, 0, 1]
*r* and *g*	Randomly generated *n* × *n* size ternary matrices
*msg_i_*	The message of a member *m_i_* for encryption
*C*	Cryptotext
$G_{\mathrm{pub}}^{\mathrm{init}}$	Initial group public key
*G_pub_*	The group public key
*Prm_i_*	The private key for member $\;m_{i}$
*PID_i_*	The pseudo ID of *i*-th member $\;m_{i}$
*k*	The number of pseudo IDs, *i* ∈ [1, k]
*Enc (·)*	The encryption operation of proposed scheme using group public key
Dec (·)	The decryption operation of proposed scheme using private key of $m_{i}$ member
*T_i_*	The timestamp in *i*-th member $\;m_{i}$
*L_i_*	The location of *i*-th member $\;m_{i}$
*D_i_*	The member’s matrix key data for group key generation

**Table 2:** PID-data table.

PID	*p*	*q*	*n*
PID_0_, PID_1_, PID_k_	*p*_0_	*q*_0_	*n*_0_
PID_10_, PID_11_, PID_1k_	*p*_1_	*q*_1_	*n*_1_
PID_k0_, PID_k1_, PID_kk_	*p*_k_	*q*_k_	*n*_k_

**Table 3:** Measurement of cryptographic operations (proposed method).

Notation	Operation	Time (μs)
*T_add_*	Matrices addition	0.5
*T_A×A_*	Matrices multiplexing	1.6
*T_A×Num_*	Multiplication matrix to number	0.5
*T_mod_*	Modular operation	0.75

**Table 4:** Measurement of cryptographic operations (PBC-based methods).

Notation	Operation	Time (ms)
*T_r_*	Bilinear pairing	3.65
*T*_0_	The exponentiation operation in multiplicative group *G*_0_	0.85
*T_T_*	The exponentiation operation in multiplicative group *G_T_*	45
*N_c_*	The number of attributes in the ciphertext	10
*N_d_*	The total number of dynamic attributes	10

**Table 5:** Computation cost.

Schemes	Key Generation	Message Encryption	Message Decryption
Xia et al. [19]	(3 + *N_d_*) *T*_0_ = 8.5 ms	(3*N_c_* + 1) *T*_0_ + *T_T_* = 30.35 ms	*T_r_* = 3.65 ms
Huang et al. [21]	(4 + 2*N_d_*) *T*_0_ = 20.4 ms	3*T*_0_ + *T_T_* = 6.55 ms	*T_r_* = 3.65 ms
Our method	*T_mod_* = 0.75 μs	*T_A × A_*+ *T_add_* + *T_mod_* = 2.85 μs	2T_A × A_ + 3T_mod_ = 6.42 μs

**Table 6:** Computation and communication cost of group key generation protocols.

Parameters	SEGKA	FCRGK	KCRT	NTRU	EGKM	VGKM	2SGKM
Computation Cost (TA or GM)	O (*n* ˄ 2) (+ nAdd + nMult)	O (*n*) (xor + Add + Mult)	O (log _τ_ *n*) (+ Add + Mult + EEA)	O (*n*) (Mult + Add + Div + EEA)	O (*n*) (Mult + Add + Div + EEA)	O (1) (Add or Sub)	O (1) (Mult + mod)
Computation Cost (User)	(+ nAdd + nMult	1mod + 1xor	1mod + 1xor	(2 Mult + 1Add + 1mod + 1EEA)	(2Mult + 1exp + 1mod + 1EEA)	1 mod	-
Storage Complexity (user)	1	2	O (log _τ_ *n*)	4	3	2	1
Storage Complexity (TA or GM)	2	4*n* *+* 1	2*n* − 1	2*n* + 7	2*n* + 5	4*n* + 3	2
Communication Complexity	1 broadcast	1 broadcast	1 broadcast	n	n	1 broadcast	1 broadcast

**Table 7:** Simulation parameters.

Parameter	Value
Environment	NS-3.29
MAC Protocol	802.11p (WAVE)
Wifi Physical Data Rate Mode	OFDM of Rate 6 Mbps, BW 10 MHz
Communication type	V2V communication
Transmission Range	250–300 m
Number of Vehicles	11–60
Vehicle speeds	10 m/s, 20 m/s, 40 m/s, 50 m/s
Mobility Model	Highway
Simulation area	1000 m × 50 m
Simulation time	100 s
Message size	200 bytes
